# Clinical characteristics and molecular epidemiology of invasive *Streptococcus agalactiae* infections between 2007 and 2016 in Nara, Japan

**DOI:** 10.1371/journal.pone.0240590

**Published:** 2020-10-19

**Authors:** Nobuyasu Hirai, Kei Kasahara, Ryuichi Nakano, Yoshihiko Ogawa, Yuki Suzuki, Miho Ogawa, Naokuni Hishiya, Akiyo Nakano, Sadahiro Ichimura, Hisakazu Yano, Masahide Yoshikawa

**Affiliations:** 1 Department of Pathogen, Infection and Immunity, Nara Medical University, Kashihara, Nara, Japan; 2 Center for Infectious Diseases, Nara Medical University, Kashihara, Nara, Japan; 3 Department of Microbiology and Infectious Diseases, Nara Medical University, Kashihara, Nara, Japan; 4 BML Biomedical Laboratories R&D Center, Inc., Kawagoe, Saitama, Japan; University of Western Australia, AUSTRALIA

## Abstract

Invasive *Streptococcus agalactiae* (GBS) infections are increasingly common among neonates and the elderly. Therefore, GBS surveillance for better antibiotic treatment and prophylaxis strategies are needed. We retrospectively evaluated the clinical aspects of invasive infections and the phenotypic and genetic diversity of infectious isolates from Nara, Japan, collected between 2007 and 2016, by using information from hospital records. GBS strains collected from the blood and cerebrospinal fluid cultures were evaluated for capsular types, multi-locus sequence typing (MLST), antibiotic susceptibility, antibiotics resistance gene, and pulsed-field gel electrophoresis. Forty GBS isolates (10 from children and 30 from adults) were analyzed, and the distribution of molecular serotype and allelic profiles varied between children and adults. We found the rates of early-onset disease in neonates with birth complications to be higher than that of previous reports, indicating that there could be relevance between complications at birth and early-onset disease. Standard antibiotic prophylaxis strategies may need to be reconsidered in patients with birth complications. In adults, the mean age of the patients was 68 years (male: 63%). Primary bacteremia was the most common source of infection. In the neonates, six had early-onset diseases and four had late-onset diseases. The most frequently identified strains were molecular serotype Ia ST23 (40%) and molecular serotype Ib ST10 (20%) in children and molecular serotype Ib ST10 (17%), molecular serotype VI ST1 (13%), and molecular serotype V ST1 (13%) in adults. Levofloxacin-resistant molecular serotype Ib strains and molecular serotypes V and VI ST1 were common causes of GBS infection in adults but were rarely found in children. Furthermore, pulsed-field gel electrophoresis in our study showed that specific clone isolates, that tend to have antibiotics resistance were widespread horizontally for a decade. Continuous surveillance and molecular investigation are warranted to identify the transmission route and improve antibiotic treatment strategies.

## Introduction

*Streptococcus agalactiae*, known as Lancefield group B *Streptococcus* (GBS), is a Gram-positive coccus species of the human gastrointestinal and genitourinary flora, and causes severe diseases, such as bacteremia, chorioamnionitis, and pneumonia [1]. Early- and late-onset GBS infections in infants occur within or after the first week of life, respectively. Most infections occur in the first three months, with high mortality and morbidity rates [2]. The Centers for Disease Control and Prevention recommends a strategy based on intrapartum chemoprophylaxis for pregnant women to lower the risk of GBS infections in infants [3].

The incidence of GBS infections is increasing in elderly patients with underlying medical conditions, such as malignancies, diabetes, and liver diseases [4]. GBS infections commonly manifest in adults as skin tissue infections, bacteremia without any obvious focus (primary bacteremia), and pneumonia. Endocarditis and meningitis have also been observed [5]. Invasive GBS infection, especially primary bacteremia, has a high mortality rate of more than 10% [6, 7].

Penicillin is the first choice for the prevention and treatment of GBS infections. Clindamycin and erythromycin are recommended as second choices for prevention in cases with penicillin allergy [8]. In 2008, GBS with reduced penicillin susceptibility was reported in Japan [9]. Moreover, levofloxacin- and clindamycin-resistant isolates have been increasingly reported in recent years [10, 11]. These reports indicate an increasing prevalence of multidrug-resistant GBS. Therefore, the choice of antibiotics for the prophylaxis and treatment of GBS infections, especially levofloxacin and clindamycin, need some reconsideration [12].

Several virulence factors are involved in the adhesion and invasion of pathogens, as well as evasion from the host immune system [13]. Moreover, the serotype and multi-locus sequence type (MLST) has been reported to be associated with the presence of virulence factors [14]. GBS can be classified into at least 10 different serogroups. A recent study reported that serotypes Ib, III, and V were those most frequently isolated in Japan [6]; however, the specific factors were not identified.

This retrospective study aimed to evaluate the clinical aspect of invasive GBS infections, and the prevalence of widespread strains in children and adults to enable the development of appropriate antibiotic treatment strategies.

## Materials and methods

### Invasive GBS cases and strains

Invasive GBS infections were defined as cases, in which GBS was isolated from blood and spinal fluid cultures. The study was performed retrospectively at the Nara Medical University Hospital for the period between June 2007 and December 2016. The medical records of patients who underwent treatment during this time were reviewed. Clinical data collected included the age, sex, date of culture, Pitt bacteremia score, source of infection, Charlson Comorbidity Index, antibiotic agents used, general anesthesia used, chemotherapy, radiation therapy, and use of immunosuppressive agents, such as glucocorticoids, administered within 30 days of the onset of bacteremia, and outcomes [15]. This study was approved by the Ethics Committee of the Nara Medical University (Project identification code: No. 1465).

### GBS identification and molecular serotype determination

The GBS strains were collected between 2007 and 2016 from the blood and spinal fluid cultures of neonates and adults, and identified using the VITEK2 system (bioMérieux, Japan). All strains were stored in sterile water, that contained 40% glycerol at -80°C until use. DNA extraction was performed using the Cica Geneus DNA Extraction reagent (Kanto Chemical Co., Inc. Tokyo, Japan); the supernatant was used for PCR. The identity of the strains was confirmed through PCR by amplifying the GBS-specific *dltS* gene region [16]. Molecular serotypes were identified using two sets of multiplex PCR reactions without molecular serotype IX (Table 1). The PCR cycling conditions were as follows: initial denaturation at 94°C for 5 min, followed by 35 cycles of denaturation at 94°C for 1 min, annealing at 49.5°C and 60°C for 1 min for the first and second sets, respectively, and an extension at 72°C for 1 min. The final extension was performed at 72°C for 5 min [16]. PCR amplicons were separated using 1.0% agarose gel. After staining with ethidium bromide, images were recorded under UV illumination. Isolates, including molecular serotype IX, which were not amplified with any of the primers, were classified as non-typeable (NT).

**Table 1:** Target gene, primer, and probe sequences for GBS molecular serotype determination and antibiotic resistant genes.

Primer name	Sequence (Forward)	Sequence (Reverse)	Target gene	Amplicon Size (bp)	Reference
Ia	GGTCAGACTGGATTAATGGTATGC	GTAGAAATAGCCTATATACGTTGAATGC	cps1aH	521 and 1,826	[16]
Ib	TAAACGAGAATGGAATATCACAAACC	GAATTAACTTCAATCCCTAAACAATATCG	cps1bJ	770	[16]
II	GCTTCAGTAAGTATTGTAAGACGATAG	TTCTCTAGGAAATCAAATAATTCTATAGGG	cps2K	397	[16]
III	TCCGTACTACAACAGACTCATCC	AGTAACCGTCCATACATTCTATAAGC	cps1a/2/3I	1826	[16]
IV	GGTGGTAATCCTAAGAGTGAACTGT	CCTCCCCAATTTCGTCCATAATGGT	cps4N	578	[16]
V	GAGGCCAATCAGTTGCACGTAA	AACCTTCTCCTTCACACTAATCCT	cps5O	701	[16]
VI	GGACTTGAGATGGCAGAAGGTGAA	CTGTCGGACTATCCTGATGAATCTC	cps6I	487	[16]
VII	CCTGGAGAGAACAATGTCCAGAT	GCTGGTCGTGATTTCTACACA	cps7M	371	[16]
VIII	AGGTCAACCACTATATAGCGA	TCTTCAAATTCCGCTGACTT	cps8J	282	[16]
dltS	AGGAATACCAGGCGATGAACCGAT	TGCTCTAATTCTCCCCTTATGGC	dltS	952	[17]
adhP	GGTGTGTGCCATACTGATTT	ACAGCAGTCACAACCACTCC	*adhP*	498	[17]
pheS	ATATCAACTCAAGAAAAGCT	TGATGGAATTGATGGCTATG	*pheS*	501	[17]
Atr	ATGGTTGAGCCAATTATTTC	CCTTGCTCAACAATAATGCC	*atr*	501	[17]
glnA	AATAAAGCAATGTTTGATGG	GCATTGTTCCCTTCATTATC	*glnA*	498	[17]
sdhA	AACATAGCAGAGCTCATGAT	GGGACTTCAACTAAACCTGC	*sdhA*	519	[17]
glcK	GGTATCTTGACGCTTGAGGG	ATCGCTGCTTTAATGGCAGA	*glcK*	459	[17]
Tkt	ACACTTCATGGTGATGGTTG	TGACCTAGGTCATGAGCTTT	*tkt*	480	[17]
ermA	AACTTGTGGAAATGAGTCAACGG	CAGAATCTACATTAGGCTTAGGG	*ermA*	375	[19]
ermB	ATTGGAACAGGTAAAGGGCG	GAACATCTGTGGTATGGCG	*ermB*	442	[19]
mefA	AGTATCATTAATCACTAGTGC	TTCTTCTGGTACTAAAAGTGG	*mefA*	345	[19]
gyrA	GGTTTAAAACCTGTTCATCGTCGT	GCAATACCAGTTGCACCATTGACT	*gyrA*	407	[20]
parC	CCGGATATTCGTGATGGCTT	TGACTAAAAGATTGGGAAAGGC	*parC*	403	[20]

### Multi-locus sequence type and clonal complex analysis

MLST determination PCR was performed using seven housekeeping genes (*adhP*, *atr*, *glcK*, *glnA*, *pheS*, *sdhA*, and *tkt*), as described in previous reports (Table 1) [17]. The reaction conditions were denaturation at 94°C for 1 min, primer annealing at 55°C for 45 s, and extension at 72°C for 1 min for 30 cycles. The PCR products were purified using a QIAquick PCR purification kit (Qiagen, Hilden, Germany), and this was followed by DNA sequencing using an ABI BigDye Terminator v3.1 cycle sequencing kit (Applied Biosystems, Foster City, CA, USA) and an ABI3730xl analyzer (Applied Biosystems). The ST and clonal complex (CC) of each isolate were determined using the GBS MLST database (http://pubmlst.org/sagalactiae/) and eBURST V3 (http://eburst.mlst.net/v3/enter_data/single/).

### Antibiotic susceptibility testing

Minimum inhibitory concentrations (MIC) were determined using the broth microdilution method (frozen plate ‘Eiken’; Eiken Chemical Co., Ltd., Tokyo, Japan), according to the Clinical and Laboratory Standards Institute (CLSI) guidelines. The susceptibility to penicillin, ampicillin, cefazolin, ceftriaxone, meropenem, erythromycin, azithromycin, clarithromycin, levofloxacin, clindamycin, and vancomycin was determined using a broth dilution method, that conformed to the standards of the CLSI. A bacterial suspension containing 5 × 10^5^ CFU of each strain was inoculated into each well for 24 h at 35°C, in accordance with the guidelines of the CLSI M100-S25 (January 2015) [18]. *Streptococcus pneumoniae* ATCC 49619 was used as the quality control strain for the antibiotic susceptibility tests. The susceptibility of *S*. *agalactiae* to these antimicrobial agents was classified based on the CLSI breakpoint criteria, as susceptible (S), intermediate (I), and resistant (R).

### Analysis of antibiotic-resistant genes

The strains were all tested for the presence of *ermB*, *ermA*, and *mefA* using multiplex PCR (Table 1). The PCR cycling conditions for amplification of these three genes comprised an initial denaturation step at 94°C for 5 min, followed by 35 cycles of denaturation at 94°C for 30 s, annealing at 50°C for 30 s, and elongation at 72°C for 1 min. The final extension was performed at 72°C for 10 min [19]. PCR amplicons were separated using 1.0% agarose gel. After mixing with EZ-VISION ONE (Mitsubishi Corporation Life Sciences Limited, Tokyo, Japan), images were recorded under UV illumination.

PCR amplification and DNA sequencing of the *gyr*A and *par*C genes were performed using primers, as described in a previous report (Table 1) [20, 21]. All of the resistant isolates had amino acid changes within the quinolone resistance-determining regions (QRDR) of *gyrA* and *parC*, compared to the reference sequence. *Streptococcus agalactiae* strain 2603V/R (ATCCBAA-611; GenBank accession number NC_004116.1) was used as the reference strain for comparative analysis. The reaction conditions were as follows: denaturation at 94°C for 1 min, primer annealing at 55°C for 45 s, and extension at 72°C for 1 min for 30 cycles. PCR products were purified using a QIAquick PCR purification kit (Qiagen, Hilden, Germany), followed by DNA sequencing using an ABI BigDye Terminator v3.1 cycle sequencing kit (Applied Biosystems, Foster City, CA, USA) and an ABI3730xl analyzer (Applied Biosystems).

### Pulsed-field gel electrophoresis

We conducted pulsed-field gel electrophoresis (PFGE) for all isolates. GBS was cultured in sheep’s blood agar (Nissui Pharmaceutical Co.) at 35°C for 24 h. Some colonies were picked up from each agar plate and placed in a cell suspension buffer (Bio-Rad Laboratories, USA). An equal volume of melted 2.0% low-melting-point agarose (InCert agarose; FMC BioProducts, Rockland, Maine) was added to this suspension. The plug-embedded GBS cells were lysed with lysozyme (5,000 U/3 mL) and mutanolysin (20 U/mL) at 50°C for 3 h, as described previously [20]. The lysis solution was decanted and replaced with a solution that contained 0.4 mg proteinase K (Wako Pure Chemical Industries, Ltd., Osaka, Japan) per mL ES (0.25 M EDTA [pH 8.0], 1% Sarkosyl) and kept at 50°C for 24 h. The ES solution was decanted, and the plugs were washed in TE buffer for 20 min at room temperature. For restriction of endonuclease digestion, the plugs were incubated in an enzyme restriction buffer for 1 h at room temperature to remove the EDTA. Chromosomal DNA was digested by incubation with *Sma*I (100 U/mL) at 30°C overnight. PFGE was performed with 1% agarose and 0.5× TBE buffer with pulse times of 4–16 s at an angle of 120° at 6.0 V/cm and 14°C for 18 h using the CHEF Mapper (Bio-Rad Laboratories, USA). Cluster analysis was performed using GelCompar II v.3.0 (Applied Maths, Sint-Martens-Latem, Belgium) by applying the unweighted-pair group method using arithmetic averages. Strains that showed at least 80% similarity with the PFGE profile were deemed to belong to the same cluster [22].

## Results

### Clinical manifestations

A total of 40 GBS isolates (10 from neonates and 30 from adults) were collected. Thirty-eight of the isolates were from blood cultures, including four umbilical cord blood cultures, and the remaining two were from spinal fluid cultures. The clinical presentations of the isolates are shown in Table 2.

**Table 2:** Clinical presentations of the 40 GBS isolates used in the study.

Variable	Neonates (n = 10)	Adults (n = 30)
Age, years [mean±SD]	EOD = 6; LOD = 4^a^	68±14.8
Age > 65 years [n (%)]		20 (67)
Gender (male) [n (%)]	4 (40)	19 (63)
Pitt bacteremia score [median (IQR)]		1 (0–2)
Nosocomial infection [n (%)]		6 (20)
Community-acquired infection [n (%)]		24 (80)
In-hospital mortality [n (%)]	0 (0)	5 (17)
**Comorbidities [n (%)]**		
Complications at birth	6 (60)^b^	
No complications at birth	4 (40)	
Solid-organ malignancy		13 (43)
Diabetes mellitus		9 (30)
Cardiovascular failure		8 (27)
Liver diseases		7 (24)
Kidney diseases		4 (13)
Hematologic diseases		4 (13)
Chronic pulmonary diseases		2 (7)
Dementia		2 (7)
Peptic ulcer		0 (0)
Charlson Comorbidity Index >2		22 (73)
Charlson Comorbidity Index [mean±SD]		3.5±2.6
**Source of bacteremia [n (%)]**		
Primary bacteremia	4 (40)	11 (37)
Skin tissue infection	0 (0)	8 (27)
Meningitis	3 (30)	1 (3)
Pneumonia	2 (20)	3 (10)
Arthritis	1 (10)	2 (6)
Urinary tract infection	0 (0)	3 (10)
Endocarditis	0 (0)	2 (6)
**Predisposing factors [n (%)]** ^c^		
Prior systemic antibiotic therapy		7 (23)
Any quinolone		4 (13)
Other antibiotics		3 (10)
Recent surgery		0 (0)
Chemotherapy		2 (7)
Recent intensive care unit admission		0 (0)
Central vein catheter		2 (7)
Immunosuppressant therapy		3 (10)

^a^ EOD, early-onset disease; LOD, late-onset disease.

^b^ Premature birth: 4 (EOD); Premature rupture: 1 (EOD); Emergency cesarean section: 1 (EOD).

^c^ Used within 30 days before incidence.

Among the 10 neonates, six had early-onset diseases (EOD), and four had late-onset diseases. Four of these 10 cases involved males. Primary bacteremia was the most common source of infection (four cases, 40%), followed by meningitis (three cases, 30%), and cellulitis (two cases, 20%). All the six patients with EOD had undergone challenges in the perinatal period, such as premature birth or premature rupture of the fetal membrane. In five of six EOD neonates, the mothers received antibiotics before delivery. The GBS isolates were susceptible to the antibiotics in all the cases.

Among the adults, the mean age was 68 years (42–97 years), and 19 cases (63%) involved males. The common underlying medical conditions were solid organ malignancy (43%) and diabetes (30%). Primary bacteremia was the most common source of infection (11 cases, 37%), followed by cellulitis (eight cases, 27%), pneumonia (three cases, 10%), urinary tract infection (three cases, 10%), infective endocarditis (two cases, 6.7%), and meningitis (one case, 3%).

The in-hospital mortality rate was 17% (five cases), and all of them were adults and tended to have higher Charlson Comorbidity Index (mean 4.3) and Pitt bacteremia scores than the other patients. The infection sources in these cases were pneumonia (three cases), cellulitis (one case), and primary bacteremia (one case). Among the adult cases, the mean score of the Charlson Comorbidity Index was 3.5. Among seven patients who had undergone systemic antibiotic therapy within 30 days of the onset of bacteremia (four received fluoroquinolones, two received cephalosporins, and one received aminoglycosides); four cases were resistant to levofloxacin, and two to macrolides.

### Molecular serotype and multi-locus sequence typing

The isolates comprised eight molecular serotypes. The most frequent molecular serotypes in the neonates were Ia (four cases, 40%), III (three cases, 30%), and Ib (two cases, 20%), and they did not show any tendency for either EOD or LOD. In adults, the frequent molecular serotypes were Ib (nine cases, 30%), VI (five cases, 17%), V (five cases, 17%), Ia (four cases, 13%), and III (three cases, 10%). Two strains were classified as NT. The frequent MLSTs in neonates were ST23 belonging to CC23 (four cases, 40%), and ST10 belonging to CC10 (two cases, 20%), while those in adults were ST1 belonging to CC1 (12 cases, 40%), ST10 belonging to CC10 (six cases, 20%), ST23 belonging to CC23 (four cases, 13%), and ST335 belonging to CC19 (three cases, 10%). The relationships between the molecular serotypes and MLSTs are summarized in Table 3.

**Table 3:** Molecular serotypes and MLST among the 40 GBS isolates.

Neonates								Adults									
Sequence types	10	17	19	23	27	335	Total	1	3	10	23	26	196	216	335	349	Total
Ia				4^a^			4			1	2			1			4
Ib	2^b^						2	1	1	5	2						9
II								1									1
III			1		1	1	3								3		3
IV													1				1
V								4				1					5
VI								4								1	5
VIII								1									1
NT		1					1	1									1
Total	2	1	1	4	1	1	10	12	1	6	4	1	1	1	3	1	30

^a^ EOD:3 LOD:1

^b^ EOD:1 LOD:1

### Antibiotic susceptibility

The minimum inhibitory concentrations (MICs) of antibiotics were determined for all the 40 GBS isolates (Table 4). None of the isolates were resistant to penicillin, ampicillin, cefazolin, ceftriaxone, cefepime, meropenem, or vancomycin. The MIC of penicillin was ≤0.06 μg/mL for all the studied isolates.

**Table 4:** Number of GBS isolates by the minimum inhibitory concentrations.

MIC (μg/mL)/cutoff (μg/mL)	≤0.06	0.125	0.25	0.5	1	2	4	8	16	32>	MIC_50_ (μg/mL)	MIC_90_ (μg/mL)
Penicillin/0.12	40^S^										≤0.06	≤0.06
Ampicillin/0.25	23^S^	17^S^									≤0.06	0.125
Cefazolin	5	33	1	1							0.125	0.125
Ceftriaxone/0.5	39^S^	1^S^									≤0.06	≤0.06
Meropenem/0.5	40^S^										≤0.06	≤0.06
Erythromycin/0.5	30^S^	3^S^		3^I^	1^R^	3^R^					≤0.06	0.5
Azithromycin/1	7^S^	24^S^	2^S^	1^S^		3 ^R^	2 ^R^		1^R^		0.125	2
Clarithromycin/0.5	34^S^	1^S^	1^S^	3^I^	1 ^R^						≤0.06	0.25
Levofloxacin/4				14^S^	15^S^				1^R^	10^R^	1	32>
Clindamycin/0.5	37^S^		1^S^		1^R^					1^R^	≤0.06	≤0.06
Vancomycin/1		2^S^		38^S^							0.5	0.5

MIC, minimum inhibitory concentration.

^S^ susceptible to antibiotic

^I^ intermediate

^R^ resistant to antibiotic.

### Relationship between molecular serotype and resistance encoding genes

**Fig 1:**
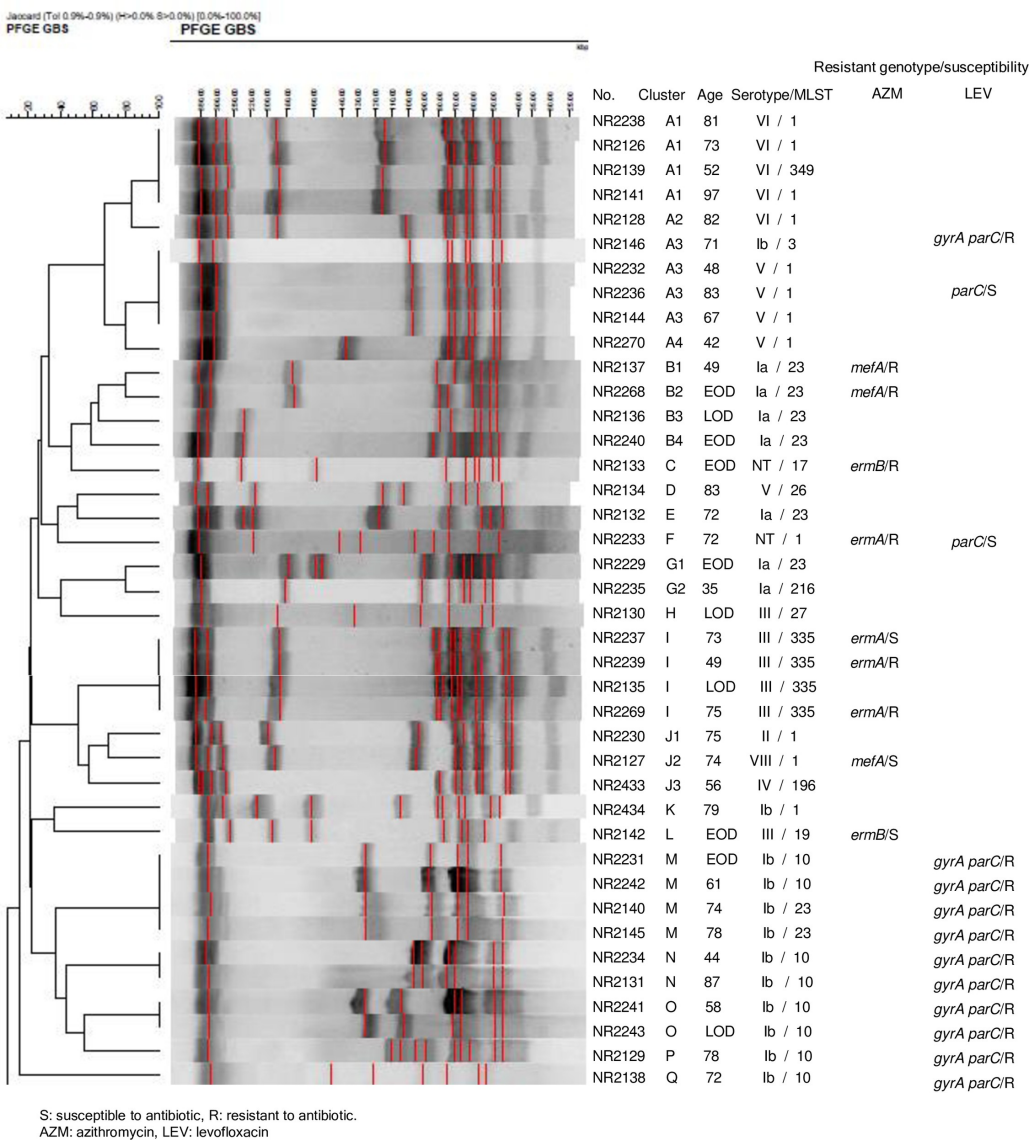
Analysis of PFGE patterns in the 40 GBS isolates.

Eleven strains (28%) were resistant to levofloxacin with high MICs (>8 μg/mL), among which two were from children and nine were from adults. The levofloxacin resistant isolates mostly carried double point mutations of DNA with inferred amino acid substitutions, including the change of Ser-81 to Leu in the *gyrA* product, and Ser-79 to Phe or Tyr in the *parC* product. We observed point mutations in 11 isolates in *gyrA* and 13 isolates in *parC*. Eleven molecular serotype Ib CC10 isolates with double mutations in *gyrA* and *parC* showed resistance to levofloxacin, although two isolates with a single mutation in just *parC* did not show resistance to levofloxacin (Fig 1). Regarding the other antibiotics, 18% (7/40), 15% (6/40), 10% (4/40), and 5% (2/40) of the strains were not susceptible to erythromycin, azithromycin, clarithromycin, and clindamycin respectively. Nine isolates had resistant genotypes: *mefA* (n = 3), *ermA* (n = 4), and *ermB* (n = 2). The relationship between macrolide resistance genes and MIC are shown in Table 5. Three of these isolates did not show high MICs for macrolide. Half of the isolates that were not susceptible to macrolides and clindamycin were isolated from neonates within the first week of life. Isolates that did not have these three resistant genotypes did not show a high MIC for macrolides. The relationships between the molecular serotype, levofloxacin-resistance genotype, and macrolide-resistant genotype are shown in Table 6. All of the levofloxacin-resistant isolates belonged to molecular serotype Ib, whereas 72% belonged to ST10. However, 67% of the macrolide-resistant genotype isolates belonged to molecular serotype Ia and III. In this study, we observed that the molecular serotype and MLST were associated with the drug-resistant genotype.

**Table 5:** Number of GBS isolates related between macrolide resistance genes and MIC.

MIC (μg/mL)		≤0.06	0.125	0.25	0.5	1	2	4	8	16
None	EM	29	2							
	CAM	30	1							
	AZM	5	24	2						
*ermA*	EM				3	1				
	CAM	2		1	1					
	AZM				1		2	1		
*ermB*	EM	1					1			
	CAM	1				1				
	AZM	1								1
*mefA*	EM		1				2			
	CAM	1			2					
	AZM	1					1	1		

MIC, minimum inhibitory concentration.

EM; erythromycin, CAM; clarithromycin, AZM; azithromycin

**Table 6:** Number of GBS isolates related between molecular serotype, levofloxacin resistance, and macrolide-resistant genes.

Serotype	Neonates					Adults				
	*gyrA*	*parC*	*mefA*	*ermA*	*ermB*	*gyrA*	*parC*	*mefA*	*ermA*	*ermB*
Ia			1					1		
Ib	2	2				9	9			
III					1				3	
V							1			
VIII								1		
NT					1		1		1	
Total	2	2	1		2	9	11	2	4	

### Pulsed-field gel electrophoresis

We obtained the PFGE banding patterns for all the 40 isolates. A dendrogram of the PFGE banding patterns is shown in Fig 1. Notably, we observed highly homologous restriction patterns among adults, with molecular serotype III belonging to ST335 and molecular serotypes V and VI belonging to ST1. The results show that isolates showing highly homologous restriction patterns tended to have the same resistant genotype and susceptibility to antibiotics; these included *mefA* in molecular serotype Ia ST23, *ermA* in molecular serotype III ST335, and *gryA* and *parC* point mutations in molecular serotype Ib ST10.

## Discussion

The opportunistic pathogen GBS is one of the leading causes of neonatal bacterial infection worldwide, commonly resulting in pneumonia, septicemia, or meningitis. In addition to neonatal diseases, GBS can also infect adults, particularly the elderly and those with underlying diseases.

In this study, we analyzed the clinical characteristics of 40 invasive GBS strains. The majority (67%) of adult patients included in the study were elderly (>65 years old); this is consistent with the findings of Farley et al. [23]. Among the adult patients, 87% had underlying diseases such as malignancy (43%) and diabetes (30%), indicating that cancer patients may be especially vulnerable to GBS infections [23, 24]. Invasive GBS infection should be considered when diagnosing patients with diabetes mellitus or a high Charlson Comorbidity Index, who show bacteremia with skin and soft tissue infections or no apparent focus of infection. The risk factors, morbidity, and source of infections did not show any remarkable changes from the last decade, as expected [4, 25]. We observed that patients who had received systemic antibiotic therapy within 30 days tended to be infected by antibiotic-resistant pathogens; these results were similar to the findings of previous reports, which suggest that the use of antibiotics results in the selection of antibiotic-resistant pathogens [26].

In this study, we observed that all patients with EOD faced challenges in the perinatal period, such as premature rupture of membrane or emergency cesarean section. It has been well known that premature birth is one of the risk factors for invasive GBS infections in neonates due to a lack or delay of GBS screening and prophylactic antibiotics [27]. Furthermore, EOD occurred despite administration of antibiotics in five of six mothers in our study. In cases with complications at birth such as premature birth and premature rupture of membranes, special attention should be provided, including performance of microbiological cultures and administration of prophylactic antibiotics for EOD. The appropriate timing and types of antibiotics for both, mothers and neonates needs further investigation.

Various studies have shown that the serotype and MLST distribution can differ based on geographical and pathophysiological settings. In Japan, Morozumi et al. studied 150 GBS isolates from invasive neonatal infections and reported that the most frequent isolates were serotype III ST19, followed by serotypes Ia ST23 and Ib ST10 [28]. They also studied 443 GBS isolates from invasive adult infections and found that the most frequent isolates were serotype Ib CC10, followed by serotype V CC1, and serotype III CC19 [6]. Moroi et al. recently reported that among 477 GBS isolates from pregnant women in Saitama, Japan, the most frequent serotype was III (22%), followed by V (18%), Ib (16%), Ia (14%), and VI (11%) [29]. eBURST analysis revealed that the predominant molecular serotypes and the CC distribution revealed in our study are generally consistent with the results of previous reports in Japan [6, 28, 29]. Notably, none of the isolates from neonatal infections belonged to molecular serotype V or VI ST1, although these were common in adult patients and in cases of maternal colonization. Lee et al. also reported that serotypes V and VI ST1 isolates were predominant in cases of maternal GBS colonization, although they rarely cause invasive GBS infections [30]. It may be speculated that ST1 GBS isolates lack the virulence factors necessary for neonatal invasion, although further study is required for confirmation. Moreover, Nagano et al. reported that ST1 isolates are capable of capsular switching and nosocomial transmission, and these factors may also play a role in the transmission of the specific ST1 clone isolates among the local population [30].

Antimicrobial resistance is a major problem for treating GBS infections. In Japan, an increase in MICs of penicillin has been reported due to mutations in *pbp2x* [9]. Although we did not evaluate mutations in *pbp2x*, none of the GBS isolates we studied here had elevated penicillin G MICs. Macrolide-resistant isolates have also been reported to be increasing worldwide [19]. Here, macrolide resistance was identified in our isolates through *mefA*, *ermA*, or *ermB*, mainly among molecular serotype Ia and III. The *erm*B genotype tends to show a high MIC for macrolides, although the isolates with macrolide-resistance genes did not always show a high MIC in our study [31]. In this study, 15% of the isolates showed azithromycin resistance, similar to previous reports [32]. We examined g*yr*A and *par*C genes, which mainly include QRDRs responsible for the fluoroquinolone-resistance phenotype [21]. In this study, only 11 isolates with double mutations in g*yr*A and *par*C showed resistance to levofloxacin, as described in previous reports [21]. All levofloxacin-resistant isolates belonged to molecular serotype Ib ST10 or ST23. Furthermore, fluoroquinolone resistance has been reported worldwide in GBS, including fluoroquinolone-resistant ST10/serotype Ib strains in Argentina and East Asia [33, 34]. As susceptibility to macrolide and fluoroquinolones is not routinely evaluated in the clinical setting, antibiotic resistant GBS strains may have already been disseminated worldwide. To our knowledge, this is the first report wherein many isolates detected from patients with invasive GBS infection for 10 years were examined by PFGE. Moreover, the findings suggested that specific isolates had widespread human transmission for at least a decade in the local community and had caused invasive infections, because we observed many isolates with highly homologous restriction patterns in PFGE with similar molecular serotype, MLST, and antibiotic susceptibility.

The results of this study are intriguing and notable in many ways; however, some limitations should be considered. First, the sample size is small, especially for neonates. This could be attributed to the fact that the samples were collected from a single hospital, and invasive GBS infection does not occur frequently. However, GBS molecular epidemiological surveillance in the region and evaluation of the difference between neonatal and adult infections were not previously undertaken. Therefore, this study provides considerable insights into the treatment and prophylaxis strategy for GBS infections in Japan. Second, it is likely that there are local variations in resistance, which limit the validity of broader generalizations. Further prospective, large-sample, local studies should be conducted in Japan to assess the prevalence of antibiotic resistance in GBS. Third, we could not draw definitive conclusions regarding the mechanism of community spread of the specific isolates because patients from whom the isolates were obtained had already been discharged and could not be followed up.

In conclusion, the findings of this study highlight the risk factors for EOD and indicate a necessity for further investigation of the antibiotic prophylaxis strategy among neonates with premature birth and rupture of membrane. Furthermore, we identified the differences and tendencies of molecular serotypes, MLST, and antibiotic susceptibility between neonates and adults. In both neonates and adults, we identified macrolide-resistant molecular serotypes Ia and III and the levofloxacin-resistant molecular serotype Ib that have already been disseminated worldwide. These antibiotic-resistant isolates must be considered when selecting an appropriate antibiotic treatment. Furthermore, we used PFGE to show that specific clones, which have antibiotics resistance genes were widespread horizontally in our local area for at least a decade. Thus, continuous surveillance and appropriate antibiotic treatment are necessary to prevent, treat, and control the spread of antibiotic-resistant infections.

## Supporting information

S1 Raw images (PDF)
