# Supplementary figures and images for: Clinical characteristics and molecular epidemiology of invasive Streptococcus agalactiae infections between 2007 and 2016 in Nara, Japan
Source: PLoS One. 2020 Oct 19;15(10):e0240590. doi: 10.1371/journal.pone.0240590 (PMC7571711; doi:10.1371/journal.pone.0240590)

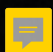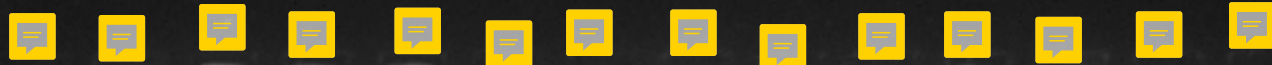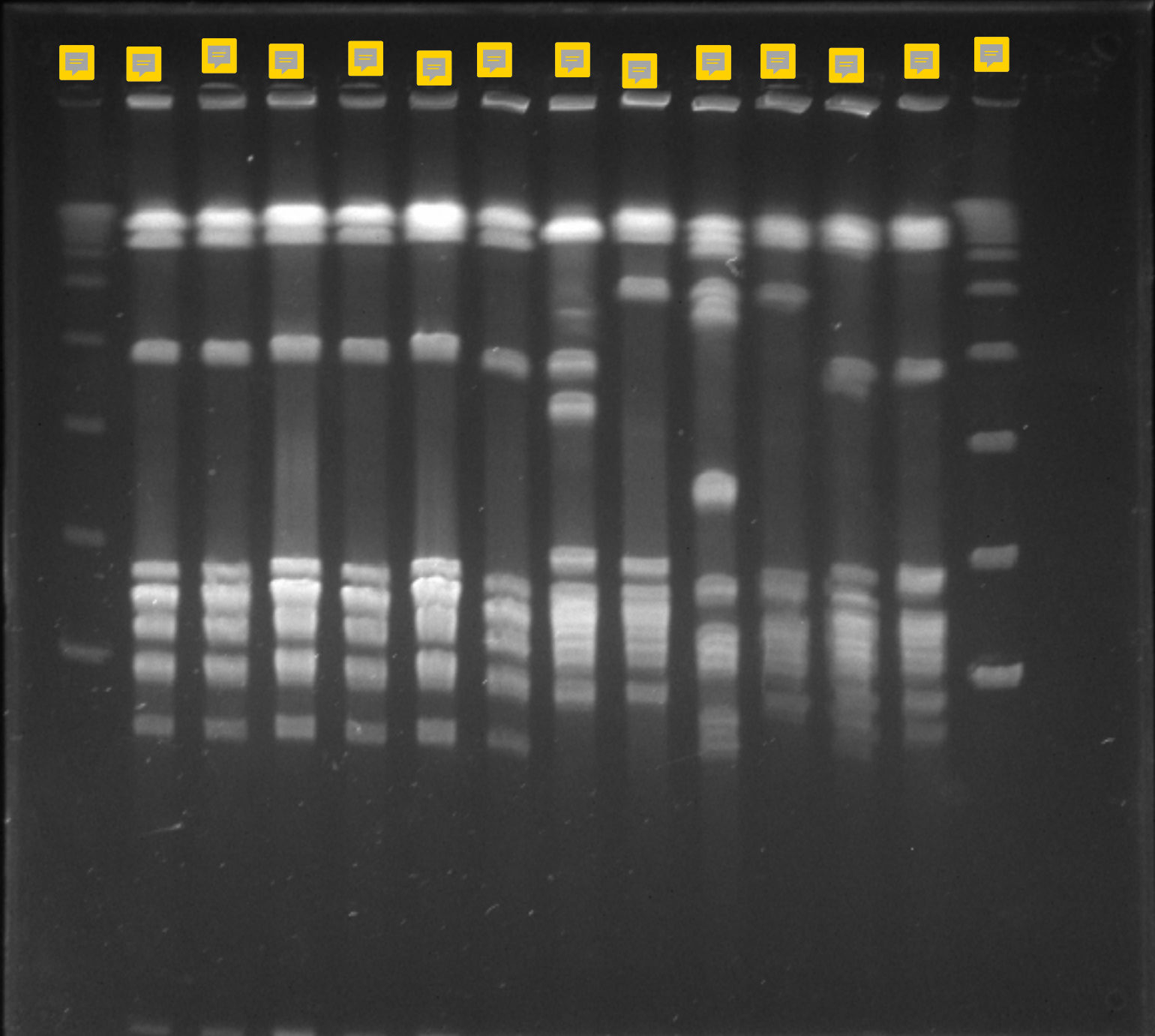

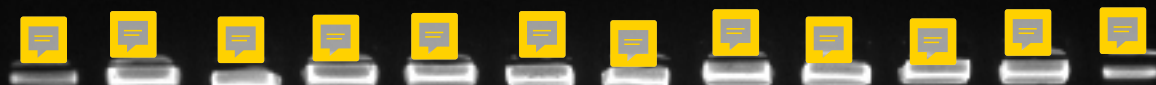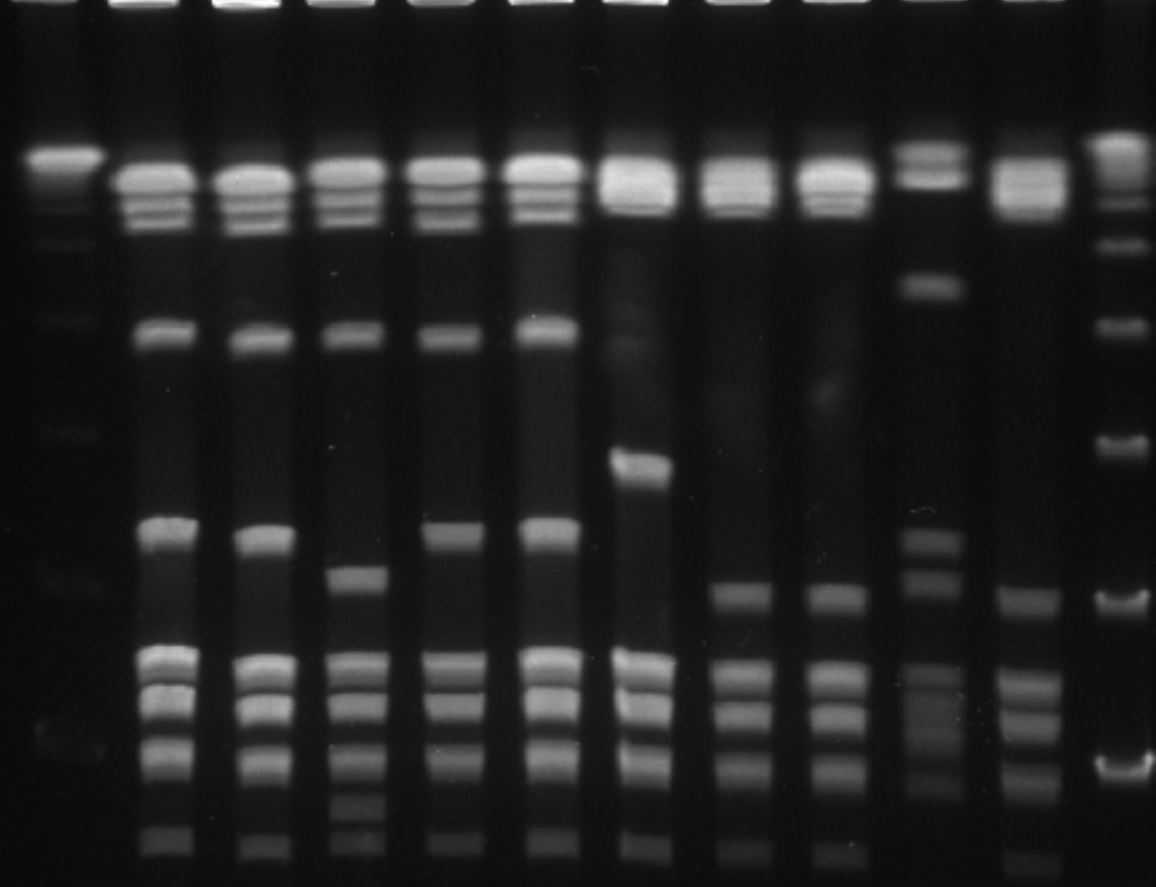

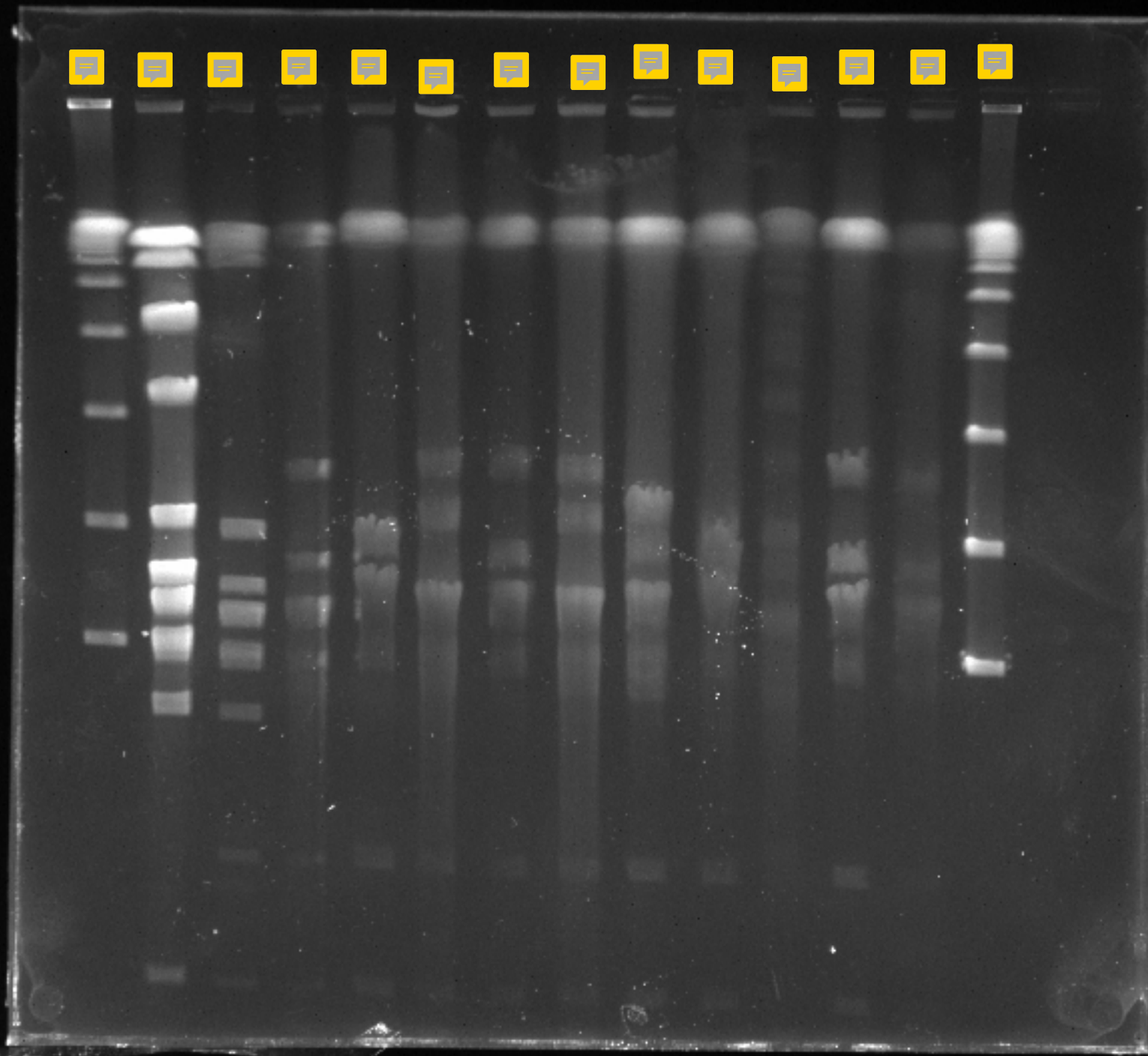

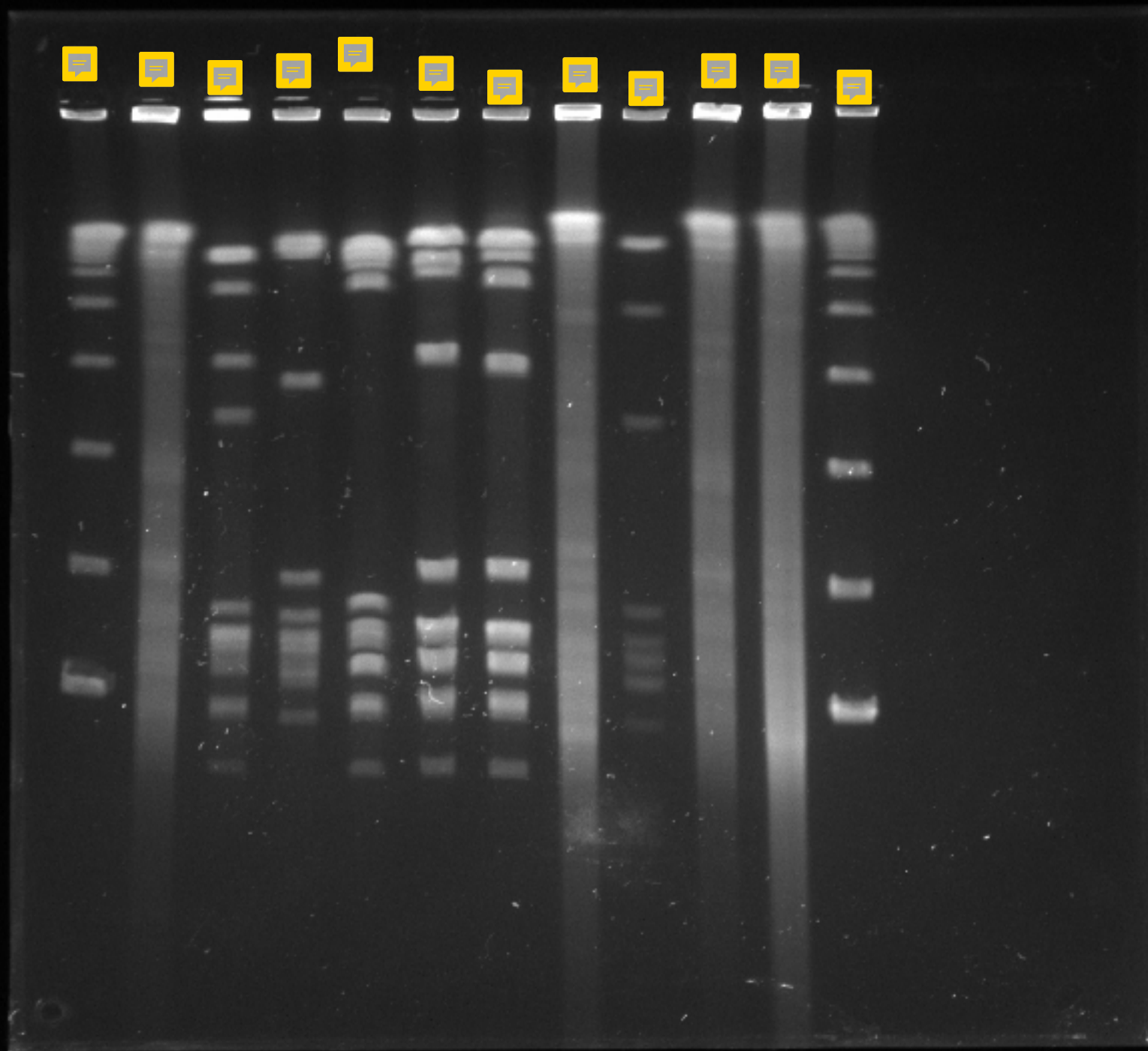

Supplement: S1 Raw images — (PDF) [file pone.0240590.s001.pdf]
